# Long Working Hours and Risk of Cardiovascular Disease

**DOI:** 10.1007/s11886-018-1049-9

**Published:** 2018-10-01

**Authors:** Marianna Virtanen, Mika Kivimäki

**Affiliations:** 1 Department of Public Health and Caring Sciences, Uppsala University, Box 564, 751 22 Uppsala, Sweden; 2 Stress Research Institute, Stockholm University, Stockholm, Sweden; 3 Department of Epidemiology and Public Health, University College London, 1-19 Torrington Place, London, WC1E 7 HB UK; 4 Department of Public Health, Clinicum, University of Helsinki, Helsinki, Finland

**Keywords:** Working hours, Cardiovascular, Ischemic heart disease, Stroke, Meta-analysis, Review

## Abstract

**Purpose of Review:**

To summarize the evidence on the relationship between long working hours and cardiovascular disease, such as coronary heart disease and stroke.

**Recent Findings:**

Large-scale meta-analyses with published and individual participant observational data on more than 740,000 men and women free of cardiovascular disease report a link between long working hours (≥ 55 h a week) and the onset of cardiovascular events. Our meta-analytic update of summary evidence suggests a 1.12-fold (95% CI 1.03–1.21) increased risk associated with coronary heart disease and a 1.21-fold (95% CI 1.01–1.45) increased risk of stroke, although the evidence is somewhat inconsistent and the possibility of residual confounding and bias cannot be ruled out. Few studies have examined the mechanisms which may be stress-related, behavioral, or biological. The recent pooled analyses suggest that increased cardiac electric instability and hypercoagulability might play a role.

**Summary:**

The evidence that long working hours are a risk factor for cardiovascular disease is accumulating and suggests a small risk. Studies on the effects of long working hours in high-risk populations and those with pre-existing cardiovascular disease, mechanistic research, and intervention studies are needed to advance this research field.

## Introduction

In the early 1800s, the industrial working week was 14 to 16 h a day, 6 days a week. Since then, enormous reductions in working hours have taken place as a result of increased efficiency and productivity, collective bargaining mainly via trade unions, and progressive legislation [1]. However, in modern society, working time is no longer limited to hours spent at the workplace. In many occupations, work can be done at any time and in any place. An increasingly common opinion is that high demands at work result in insufficient time to get work done within a standard 7- to 8-h workday. For low-wage blue-collar employees, long working hours may comprise two or more contemporaneous part-time jobs.

Globally, the longest annual average working hours are those in Mexico, Costa Rica, and South Korea [2], although a work schedule that has become pervasive in Chinese companies is commonly referred to as ‘996’: working from nine in the morning to nine in the evening, 6 days a week [3]. In Europe, the average number of working hours seems to be decreasing. However, a detailed analysis of extreme working hours shows polarization with an increasing proportion of the workforce both working very long hours and short hours [4]. This specific pattern was observed at least in Europe and North America.

In this review, we summarize the evidence provided by prospective studies on long working hours and cardiovascular disease (CVD), the leading cause of death globally. We also include studies that have addressed potential mechanisms linking long working hours with CVD risk and discuss limitations in the present evidence, prospects for future studies, and implications for clinical practice.

## Long Working Hours and Cardiovascular Disease: The Current Evidence

Numerous reviews have suggested that long working hours may have adverse effects on health [5–14]. Particular attention has been paid to cardiovascular diseases (CVD) stemming from the observation in Japan of ‘karoshi’—death from overwork [15]. However, to assess causation, the most convincing evidence should come from randomized controlled trials. Regarding CVD, we are not aware of any studies that have randomized participants in terms of working hours to assess the effects on CVD incidence or progression. Therefore, the evidence available relies on observational data with known limitations, as will be discussed later.

The first systematic review and meta-analysis of observational studies on the association with coronary heart disease (CHD) was published in 2012 [16]. The meta-analysis included only published studies (*n* = 12), of which 7 were case-control studies, 4 prospective, and 1 a cross-sectional study. This suggested an overall relative risk of 1.59 (95% CI 1.23–2.07) associated with long working hours. An analysis restricted to prospective studies found a relative risk of 1.39 (95% CI 1.12–1.72) while the case-control studies indicated an odds ratio of 2.43 (95% CI 1.81–3.26) for long working hours. The authors considered a major limitation among the studies to be the inconsistent assessment of exposure (long working hours) as well as problems related to publication bias and case-control design (recall bias among CHD cases).

In the Individual-Participant-Data Meta-analysis in Working Populations (IPD-Work) Consortium [17,18••], these limitations were addressed by collecting both published and unpublished data from prospective cohort studies and by carefully harmonizing the exposure (long working hours) and cardiovascular outcomes to be as consistent across studies as possible. The IPD-Work Consortium had already published a meta-analysis on perceived work stress and CHD in 2012 [17]. An individual participant meta-analysis on long working hours and the incidence of CHD and stroke from IPD-Work was published in 2015 [18••]. It included studies from the USA, Europe (the UK, Northern Ireland, Germany, Belgium, the Netherlands, Denmark, Sweden, Finland), Israel, and Australia. In 22 cohort studies and 598,470 participants for the analysis of CHD and 14 cohort studies and 520,925 participants for the analysis of stroke, hazard ratios for working 55 h or more a week, compared to a standard 35–40 h working week, were 1.13 (95% CI 1.02–1.26) for CHD and 1.33 (95% CI 1.11–1.61) for stroke. A dose-response relationship (increasing risk associated with increasing working hours in full-time employees) was found for stroke but not for CHD. Sub-group analyses, multivariable adjustment for other risk factors, and analyses stratified by the method of stroke ascertainment suggested that the excess risk of stroke was robust. Reverse causality bias was addressed by excluding cases that occurred during the first 3 years of follow-up—no evidence was found to suggest that the association was attributable to reverse causation.

Since the IPD-Work meta-analysis was published, at least two independent large-scale studies have examined the association between long working hours and cardiovascular disease. In these studies, responses of 145,861 to 199,035 employees to the Danish Labour Force Survey in 1999 to 2013 were linked to records of hospitalizations and deaths from national registers until 2014 [19•,^20^•]. With 35–40 working hours per week as reference, the estimated rate ratio for working ≥ 55 h per week was 0.89 (95% CI 0.69–1.16) for overall stroke, 0.86 (95% CI 0.61–1.22) for ischemic stroke, and 1.33 (95% CI 0.82–2.15) for hemorrhagic stroke [19•]. The rate ratio of ischemic heart disease for > 48 compared with 32–40 weekly working hours was 1.09 (95% CI 0.96–1.24 among participants without a recorded heart disease 5 years before the survey (the rate ratio was not reported for ≥ 55 weekly working hours) [20•].

**Fig. 1:**
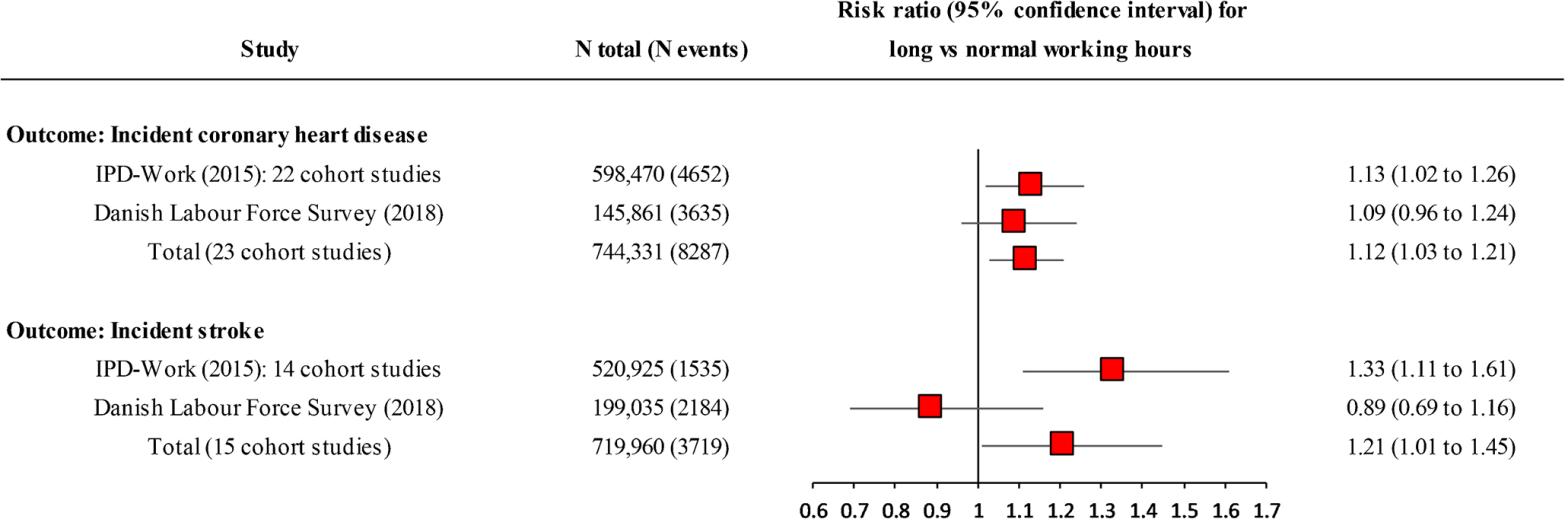
Results from random effects meta-analyses for the association between long working hours and the incidence of coronary heart disease and stroke, including the studies participating in the IPD-Work Consortium (22 cohort studies for coronary heart disease, 14 for stroke [18••]) and findings from the Danish Labour Force Survey (Hannerz et al. 2018 [19•, 20•])

Figure 1 shows the current state of evidence on the association between long working hours and the onset of CHD and stroke, in which the results from the previous systematic review and individual-participant data meta-analysis by IPD-Work published in 2015, which included 22 studies on CHD and 14 studies on stroke [18••], are supplemented with findings from the Danish Labour Force Survey [19•,^20^•], which were identified in our literature search of PubMed in June 30, 2018. The Danish studies are based on the same population-based cohort from Denmark, with an average follow-up of 7.7 years. For the CHD meta-analysis, we used the estimate in which all CHD cases 5 years before the survey were excluded instead of 1 year, as was done in the main analysis of that study [20•]. We used random effects meta-analysis (Stata 15.1) to obtain new relative risk estimates for CHD and stroke, including all original estmates from 22 CHD studies and 14 stroke studies in the analyses, to which we added the estimates reported in the Danish studies.

As shown in Fig. 1, combining the findings from IPD-Work and these new studies in a random effects meta-analysis led to a relative risk of 1.12 (95% CI 1.03–1.21) for CHD and 1.21 (95% CI 1.01–1.45) for stroke. The estimates for coronary heart disease are similar for both studies, but the point estimates of stroke from IPD-Work and the Danish Labour Force Survey appear to differ although the *I*^*2*^ statistics suggested no significant heterogeneity between the 23 and 15 meta-analyzed studies (*I*^2^ *=* 0.0%, *p* = 0.54 for CHD; *I*^2^ *=* 15.2%, *p* = 0.28 for stroke). There were methodological differences between IPD-Work and the Danish study. Stroke incidence, for example, was lower in IPD-Work (4.5 per 10,000 person-years) than in the Danish Survey (14.3 per 10,000 person-years), which might point to diagnostic differences being relevant.

In summary, the total data available from observational studies suggest small associations between long working hours and CVD outcomes. The associations seemed not to be confounded by known CVD risk factors, such as health behaviors.

## Mechanisms Linking Long Working Hours to Cardiovascular Diseases

‘Mechanisms’ refer to the pathways through which the exposure (here long working hours) has an effect on health (CVD). The particular mechanism that links long working hours to health outcomes is related to *reduced time* available for other activities besides work. Employees working long hours—because they spend *more time* at the workplace than other employees—may also be increasingly exposed to psychosocial and physical workplace hazards, such as high demands (which can also be an underlying cause of extended working hours), noise, dust, toxic chemicals, lack of natural light, and some other hazardous working conditions.

The hypothesized mechanism between long working hours and cardiovascular health, in particular, involves the potential effects associated with psychological over-activation, ‘stress’, and its impacts on the cardiovascular systems [21••,^22^] through, for example, elevation of blood pressure and heart rate, or through impaired health-related behaviors. Thus, the more distal risk factors (psychosocial) may be linked to more proximal ones (behavioral) both of which in turn may be linked to the most proximal ones (biological), forming a chain of risks that are interconnected and with which long working hours may be associated. Here, we summarize the evidence regarding psychosocial, behavioral, and biological risk factors as potential mechanisms linking long working hours to CVD risk.

## Psychosocial Mechanisms

Psychosocial factors in the etiology of CVD have been studied for several decades, commonly investigated exposures being stress at work and social isolation [21••]. Psychosocial factors also include state of mood, particularly depression, anxiety, and anger, which have been linked to an increased risk of CVD [21••,^23^]. The most commonly used formulation of work stress is ‘job strain’, a combination of high job demands and low job control, for which there is robust evidence as a risk factor for coronary heart disease and stroke [21••]. However, we are not aware of any studies that have assessed psychosocial factors at work as a mechanism between long working hours and CVD. Job strain is an unlikely link, because people who work long hours are usually in higher occupational positions and therefore more likely to have ‘active’ psychosocial work (i.e., high demands and high control). This was confirmed in the Whitehall II study of British civil servants, which found that employees working long hours reported higher job demands and greater job control than those working shorter hours [24]. In future studies, another conceptualization of psychosocial stress at work, ‘effort-reward imbalance’ [25], would be worth investigating in the context of long working hours.

A recent systematic review and meta-analysis included both published data and individual participant data and examined the association between long working hours and the onset of depressive symptoms [26]. The findings suggested a moderate-sized association in Asian countries (odds ratio [OR] = 1.50), a weak association in Europe (OR = 1.11), and no association in North American cohorts (OR = 0.97) [26]. The Whitehall II study reported higher levels of type A behavior pattern (anger, irritability, competitiveness) among those who worked long hours, but this personality characteristic did not explain the association between long working hours and CHD [24]. Thus, the current evidence on psychosocial factors as potential mechanisms between long working hours and CVD is limited and as such, does not give strong support to psychosocial factors as a mediating factor between long working hours and CVD.

A major limitation in existing studies is the lack of longitudinal assessment of long working hours and psychosocial factors over time, i.e., as time-varying exposures. However, one study from Korea used a case-crossover design and found a significant increase in average weekly working hours right before the cardiovascular event [27]. Therefore, future studies would benefit from shifting the focus from the assessment of static baseline characteristics to time-varying and triggering effects that may explain the link between long working hours and CVD.

## Behavioral Mechanisms

Established behavior-related risk factors for CVD include smoking, overweight or obesity, low physical activity, and risky alcohol use. Although cross-sectional analyses have shown that people who work long hours have higher prevalences of smoking, obesity, low physical activity, and risky alcohol use [28,29], adjustments for these as covariates have not substantially affected the association between long working hours and CVD [18••,^28^]. A recent systematic review and meta-analysis of published studies and individual participant data suggested a statistically significant, albeit modest (OR = 1.12), prospective association between long working hours and the onset of risky alcohol use [30]. Sleep disturbances, particularly short sleep—although not always lifestyle-related—have also been found to be associated with increased risk of CVD [31]. There is evidence, mainly based on cross-sectional studies, that people who work long hours sleep shorter hours, whereas the evidence on sleep disturbances is inconsistent [32]. A prospective study using the Whitehall II data found an increased likelihood of shortened sleeping hours and difficulties in initiation of sleep among those who worked long hours at baseline [32], while another prospective study did not confirm this finding [33]. The extent to which sleep explains the association between long working hours and CVD remains unknown.

In summary, current evidence does not give strong support to any single behavioral pathway linking long working hours to CVD risk. However, again, few studies have examined changes in health risk behaviors over time, which may have led to an underestimation of their contribution to CVD risk. Extensive sitting as a mechanism would be another important study topic because sitting has been suggested to increase the risk of CVD [34].

## Biological Mechanisms

Biological mechanisms refer to biological risk factors for CVD, such as elevated blood pressure, adverse lipid profile, impaired cardiac function, metabolic syndrome, diabetes, and increased inflammatory markers. To our knowledge, no studies have been published that have tested the magnitude or relative importance of biological mechanisms; instead, biological risk factors have been adjusted in the statistical models with no notable effects on the estimates [18••,^28^]. Some studies, most of them cross-sectional, have examined the association between long working hours and single biomarkers. The Whitehall II study of British civil servants, for example, observed no consistent associations between long working hours and cardiometabolic factors such as blood pressure, lipid levels, or systemic inflammation [28]. Three studies reported an association with self-reported hypertension [29,35,36] while no association was found in one prospective study [37], and still other studies have found the risk of hypertension to be lower among overtime workers than among those who work standard working weeks [38,39]. Endothelial dysfunction is observed in the early stages of atherosclerosis and is associated with increased plaque rupture, for example, in myocardial infarction. The U.S. Multi-Ethnic Study of Atherosclerosis (MESA) found no association between working hours and endothelial dysfunction, as measured by brachial artery flow-mediated dilation [40]. The evidence is also inconsistent for the association with metabolic syndrome, suggesting both positive and null findings [41,42]. A large-scale individual participant meta-analysis examined the association between long working hours and the onset of treated diabetes, reporting an association among participants with low socioeconomic status but not among those with high socioeconomic status [43].

Most studies on long working hours have examined CVD etiology, that is the role played by long working hours in the development of CVD. However, recent studies on psychological stress suggest that stress effects may actually be more pronounced in people with pre-existing cardiovascular or metabolic disease or among those at an advanced stage of developing these. One hypothesis might therefore link long working hours to stress, which in turn contributes to insulin resistance, arrhythmia, hypercoagulation, and ischemia, and cause temporary elevations in blood pressure, all of which can increase the likelihood of cardiovascular and cerebrovascular events in individuals with high atherosclerotic burden and compromised glucose metabolism [21••,^22^]. For example, the research shows stronger associations between job strain and mortality in people with pre-existing diabetes, coronary heart disease, or a history of stroke than in those free of these diseases [44]. We are not aware of studies on long working hours and recurrent CVD or mortality in employees with these cardiometabolic diseases, although the challenge in this case is that people might not be able to continue excessive working following a severe cardiovascular event.

However, evidence is accumulating on the links between long working hours and some of the triggering mechanisms. A recent IPD-Work individual participant data meta-analysis focused on long working hours and the risk of atrial fibrillation [28]. This is the most common cardiac arrhythmia and involves a high risk of developing stroke, heart failure, and dementia. One of its major etiologic risk factors is CVD, and therefore, it can also be considered as a consequence, or comorbid disease, of CVD. After adjustment for known risk factors and pre-existing CHD, the meta-analysis suggested a 1.41-fold (95% CI 1.12–1.78) increased risk of atrial fibrillation associated with working 55 h or more per week, compared to 35–40 weekly hours. This association was unchanged after exclusion of those who had had a cardiovascular event before the diagnosis of atrial fibrillation.

Irregular rhythm resulting from atrial fibrillation, by disrupting the flow of circulation, can cause blood to pool in the left atrial chamber of the heart contributing to clot formation, especially in the presence of hypercoagulability. The clot can then travel from the heart to the brain and result in a stroke [45]. We are not aware of any studies directly linking hypercoagulation in arteries to the association between long working hours and CVD. However, in agreement with this link is the observation of increased stroke risk among individuals who work long hours [18••]. In addition, a recent IPD-Work analysis provides support for increased clotting risk by reporting an association between long working hours and hypercoagulability on the venous side of the circulation, as indicated by venous thromboembolism [46]. Venous thromboembolism results from a blood clot that forms within a vein. In IPD-Work, the relative risk of venous thromboembolism for individuals working long hours compared with those working standard hours was 1.49 (95% CI 1.06–2.11). The association with deep vein thrombosis (a clot in a deep vein, usually in the leg) was stronger (relative risk 1.68, 95% CI 1.13–2.52) while the association with pulmonary embolism (a sudden blockage in a lung artery) was less robust (relative risk 1.36, 95% CI 0.77–2.38). Finally, a study from Japan reported an association between extensive overtime working and autonomic nervous system abnormalities, a further marker of stress-related mechanism that may trigger cardiac events [47].

## Conclusions and Implications

Current evidence from observational studies suggests a small association between long working hours and cardiovascular events, such as coronary heart disease and stroke. These associations seem to be consistent with no major heterogeneity between studies and with dose-response relationships observed in some studies. The observed estimates were also robust to adjustment for confounding, although residual confounding cannot be ruled out. With evidence on increased cardiac electric instability and hypercoagulability among those working long hours, there is some evidence of biological plausibility.

These findings are consistent with the Bradford Hill criteria of causality [48], which states that a cause and effect association would include temporal order (the exposure precedes the onset of disease), consistency across different studies, biological plausibility, and specificity (the association observed in a specific group of diseases). However, no evidence is available on reversibility (reversing the exposure to long working hours would reduce the disease risk), and the observed relative risk estimates are not large (i.e., > 2). At this stage, we cannot be confident about causality because there are no intervention studies or randomized control trials (RCTs) to test whether a reduction of working hours would lead to a reduction of CVD.

Today, most developed countries apply working-time regulations that allow employees to restrict the number of working hours. According to the 2003 European Worktime Directive [49], a worker’s working time should not exceed 48 h (including overtime) per week when averaged over a reference period, usually 17 weeks. The legislation is not based on research evidence of cardiovascular health only, but on a broader viewpoint of human rights, such as employees’ right to leisure time, employee well-being, and safety [50].

For future research, there is a need for a more careful assessment of mechanisms, including the use of counterfactual approaches [51], assessment of triggering effects, and consideration of new potential mediators, such as adherence to self-care and help-seeking behaviors which may also explain differences in health outcomes between those who work long hours and those who do not [52]. A further important question is how much exposure constitutes the risk of a cardiac event (months or years) and which factors might contribute to individual differences in the tolerance to working long hours. Thus, there may be moderators—which can either buffer or intensify the effects of long working hours—and which can include both personal and work-related characteristics. For example, a combination of long working hours and low job control may have a more adverse effect on health than a combination of long working hours and high job control [53]. Furthermore, physical activity may buffer an individual from the effect of long working hours on physical health, as suggested by one study on long working hours and ischemic heart disease [54]. Given the observed associations between long working hours and factors that can trigger a cardiac or cerebrovascular event in vulnerable people, more research is needed to determine whether the effects of long working hours are greater in individuals with high atherosclerotic burden or disturbed glucose metabolism than those with a healthy circulatory and metabolic system. Finally, we encourage researchers to conduct intervention studies, such as RCTs or natural experiments. Such studies would advance research into the relationship between long working hours and CVD to the next level.
